# An observation of the clinical efficacy of combining Riluzole with mannitol and hyperbaric oxygen in treating acute spinal cord injury

**DOI:** 10.12669/pjms.37.2.3418

**Published:** 2021

**Authors:** Huan-xia Li, Jing Cui, Jing-shi Fan, Jian-zhou Tong

**Affiliations:** 1 Huan-xia Li, Department of Neurosurgery, West Part, Baoding First Central Hospital, Baoding, 071000, Hebei, P.R. China; 2 Jing Cui, Department of Neurosurgery, West Part, Baoding First Central Hospital, Baoding, 071000, Hebei, P.R. China; 3 Jing-shi Fan, Department of Neurosurgery, West Part, Baoding First Central Hospital, Baoding, 071000, Hebei, P.R. China; 4 Jian-zhou Tong, Department of Neurosurgery, West Part, Baoding First Central Hospital, Baoding, 071000, Hebei, P.R. China

**Keywords:** Riluzole, acute spinal cored injury, movement function, sensory function, cytokine

## Abstract

**Objective:**

To examine the clinical efficacy of combining Riluzole with mannitol and hyperbaric oxygen therapy in treating thoracolumbar vertebral fracture-induced acute spinal cord injury (ASCI).

**Methods:**

From June 2015 to May 2018, 80 patients with thoracolumbar fractures and ASCI who were treated at Baoding First Central Hospital were selected. All patients underwent posterior laminectomy and screw fixation, and they were randomly divided into two groups using a random number table method. The control group received conventional postoperative treatment, while the experimental group was treated with riluzole combined with mannitol and hyperbaric oxygen on the basis of conventional treatment. The recovery of nerve function which included motor function and sensory function, and the changes of serum IL-6, CRP, BDNF, BFGF and other factors before treatment and four weeks after treatment of the two groups of patients were observed and evaluated.

**Results:**

After treatment, the motor function scores and sensory function scores of the two groups of patients were improved compared with those before treatment (p<0.05). Compared with the control group, the experimental group improved significantly, and the difference was statistically significant (p<0.05). The levels of IL-6, BDNF and NFGF in the experimental group were significantly lower than those in the control group (p<0.05).

**Conclusions:**

For patients with thoracolumbar fractures and ASCI undergoing laminar decompression and fixation, the comprehensive treatment plan of riluzole combined with mannitol and hyperbaric oxygen has certain advantages. Compared with the conventional therapy, it may significantly improve the movement and sensory functions of patients, relieve the inflammatory response of spinal cord, and promote recovery from the injury.

## INTRODUCTION

Having become a common emergency in the emergency service of spine surgery department,^1^ acute spinal cord injury (ASCI) is highly possible to incur a paraplegia and a bad prognosis as well.^2^ According to the prevailing therapy, the patients of thoracolumbar vertebral fracture complicating ASCI should undergo operation as early as possible to eliminate the spinal cord nerve compression and accelerate neural functional recovery of the spinal cord. However, although surgical treatment alone can reduce the overall disability rate, it has little effect on the damaged spinal cord in reducing local inflammation and promoting recovery. Other treatment methods such as hormone therapy are often needed. Other treatment methods such as hormone therapy are also necessary. In recent years, we have made some progress in applying an integrated therapeutic regime of combining Riluzole with mannitol and hyperbaric oxygen therapy to the ASCI patients having received posterior laminectomy and screw fixation operation.

Our objective was to examine the clinical efficacy of combining Riluzole with mannitol and hyperbaric oxygen therapy in treating thoracolumbar vertebral fracture-induced acute spinal cord injury (ASCI).

## METHODS

### Patient inclusion criteria

(1) The patients meet the international standards for the classification of spinal cord injuries^3^ and the standards of the British Emergency X-ray Imaging Research Institute.^4^ (2)The time from injury to treatment is less than 24h. (3) Patients with ASIA injury grades B, C, D. (4) Patients with independent ability to complete the treatment and sign the informed consent.

### Patient exclusion criteria

(1) Patients with previous spinal cord disease. (2) Patients younger than 14 years old or older than 75 years old. (3) Patients with previous history of any chronic inflammatory disease or autoimmune disease. (4)Patients administered with hormone or immunosuppressor.

### Ethical Approval

The study was approved by the Institutional Ethics Committee of Baoding First Central Hospital (No.[2020]004) Date: 04-05-2020, and written informed consent was obtained from all participants

Eighty patients with thoracolumbar fractures and ASCI who were treated at Baoding First Central Hospital from June 2015 to May 2018 were selected. Using a completely randomized design experimental method, these 80 patients were randomly divided into two groups according to the random number table method. There are 40 patients in each group. The general data of two groups were comparable without a significant difference (p>0.05).

### Therapies

All the patients underwent posterior laminectomy and screw fixation. After the operation, patients in the control group were postoperatively administered with conventional anti-inflammatory therapy and impact therapy with methylprednisolone as per hormone regime[5] which demands first IV dose of 30ms/kg within 15 minutes and then 23 hour maintenance treatment of 5.4mg / (kg h) 45min later. As for the experimental group, in addition to conventional therapy as in the control group, a combination of Riluzole with mannitol and hyperbaric oxygen was applied in accordance with following schedule: mannitol (250ml, IV, b.i.d, 7days); Riluzole (oral, 100mg, every 12h on 1d; 50mg, bid, 14days); hyperbaric oxygen (100 minutes, s.i.d, compressed for 20min to reach 2ATA, stabilized oxygen inhalation for 60min, decompressed for 20 minutes). Each cycle lasts 10 days and three days of suspension are kept between two adjacent cycles. In general, 3-4 cycles are preferable.

### Movement and sensory functions

Three experienced neurosurgeons were invited to assess the neurological functions of patients in both experimental and control groups by rating their movement and sensory functions before and four weeks after the treatment as per the scales^6^. The assessment covered sensory function (acmesthesia and light touch) and movement function. In the sensory function assessment, 28 critical points on both sides of the body were assessed in terms of acmesthesia and light touch. Each critical point was scored from 0 to 2 (o for sensory loss, one for dysfunctional sensation, and two for normal sensation). Movement function assessment was to score the myodynamia of 10 pairs of key muscles on both sides of the body as per a five-level scale (level 0: complete paralysis; level-1: without muscular contraction; level-2: muscular contraction only, but without muscular movement; level-2: limbs able to make horizontal movement but unable to resist one’s own gravity; leve- 3: limbs able to be elevated away from wound surface but unable to endure the resistance; level-4: ability of comprehensive joint motion against resistance; and level-5: normal myodynamia).

*Changes of IL-6, CRP, BDNF and BFGF in serum* Morning and fasting blood were collected from patients in both groups before and 4 weeks after the treatment. Changes in the levels of serous IL-6, CRP, BDNF and BFGF were detected using ELISA on a full-automatic microplate reader (Bio-Rad, U.S.).

### Statistical methods

The data were analyzed with SPSS 19.0. Measurement data were presented as inline±S. Intergroup difference was detected with independent-sample t-test. Intra-group comparison before and after the treatment adopted paired t-test. Rate comparison was done with χ^2^ test. A P-value <0.05 denotes a statistical difference.

## RESULTS

Before the treatment, two groups didn’t differ greatly in scores of sensory (both acmesthesia and light touch) and movement functions (p>0.05). Both groups gained a significant improvement in the scores of sensory and movement functions 4 weeks after the treatment (p<0.05), but the improvement effect in experimental group was more statistically evident (Table-I).

**Table-I:** A comparison of experimental group with control group in neurological assessment before and after the treatment (in X̄ ±S, n=40)

*Group*	*Sensation score (acmesthesia)*				*Sensation score (light touch)*				*Movement score*			

	Before*	*AfterΔ*	*t*	*p*	Before*	*AfterΔ*	*t*	*p*	Before*	*AfterΔ*	*t*	*p*
Experimental groupΔ	60.72±7.13	99.74±7.93	23.14	0.01	55.73±7.17	105.21±10.70	26.75	0.00	46.72±5.81	89.76±7.18	29.47	0.01
Control groupΔ	59.89±6.75	82.11±7.74	13.68	0.03	54.78±6.52	84.47±7.86	18.39	0.00	46.57±5.64	70.23±6.55	17.31	0.01
t	0.53	10.06			0.62	25.45			0.12	12.71		
p	0.59	0.00			0.53	0.00			0.90	0.00		

* p>0.05,

Δ p<0.05

After= 4 weeks after the treatment.

In both groups, levels of IL-6, CRP, BDNF and BFGF were higher than normal values before the treatment was initialized but not significantly higher (p>0.05). Four weeks after the treatment was applied, IL-6, CRP, BDNF and BFGF were significantly improved in both experimental and control groups when compared with that before the treatment (p<0.05). Among those indicators, IL-6 was significantly improved in the experimental group (p<0.05), but difference in CRP was so evident (p>0.05) (Table-II). The experimental group also had significantly improved BDNF and BFGF when compared with the control group after the treatment as applied (p<0.05) (Table-III).

**Table-II:** A comparative analysis of IL-6 and CRP levels before and after the treatment in both groups ( X̄±S, n=40)

*Inflammatory factor*	*IL-6(ng/L)*				*CRP (mg/L)*			

*Group*	Before*	*AfterΔ*	*t*	*p*	Before*	After*	*t*	*p*
Experimental groupΔ	15.10±5.23	8.97±2.24	6.81	0.01	3.47±0.55	2.21±1.12	6.39	0.00
Control groupΔ	15.25±4.28	11.12±1.74	5.65	0.03	3.73±1.03	2.35±1.33	5.19	0.00
t	0.14	4.79			1.41	0.52		
p	0.89	0.00			0.16	0.61		

* p>0.05,

Δ p<0.05

After= 4 weeks after the treatment.

**Table-III:** A comparative analysis of BDNF and BFGF before and after the treatment in both groups ( X̄ ±S, n=40)

*Cytokine*	*BFGF(ng/ml)*				*BDNF(ng/ml)*			

*Group*	Before*	*AfterΔ*	*t*	*p*	Before*	*AfterΔ*	*t*	*p*
Experimental groupΔ	16.52±1.23	1.05±0.11	79.22	0.00	7.65±0.43	2.03±0.25	71.46	0.00
Control groupΔ	16.37±2.06	2.33±0.78	40.31	0.00	7.59±0.51	2.55±0.80	33.59	0.00
t	0.39	10.28			0.57	3.92		
p	0.65	0.00			0.52	0.02		

* p>0.05

Δ p<0.05

After= 4 weeks after the treatment.

## DISCUSSION

ASCI is a common complication of thoracolumbar vertebral fracture.^7^ Once ASCI is incurred, sensory and movement functions below the injured plane would be impaired, which brings in great limitation to the limb functions and self-care ability of patients. Furthermore, long-term bedridden patients are more susceptible to such complications as urinary system infection, hypostatic pneumonia, amyotrophy, and bedsore. All those will impose heavy economic and psychological burden on the patients and their family.^8^

There are two factors underlying the occurrence of ASCI. 9 One of them is direct violent injury, the more common is thoracolumbar fracture. While the other is secondary injury resulting from ischemia, that is, the internal metabolic changes, biological changes, and pathophysiological changes that occur after spinal cord injury. The spinal cord has relatively few collateral circulation and has poor tolerance to ischemia and hypoxia. Therefore, spinal cord ischemia, edema, and even necrosis easily occur after injury.^10^ Secondary injury usually merits more attention than direct violence-induced injury^11^, because the former could be alleviated through operation and fixation but the latter needs further combination of drugs in addition to the operative therapy.

At present, methylprednisolone is preferred to bring impact to the post-operative spinal cord injury. It has become the first-line medication recommended by the guidelines. This agent could lower the number of inflammatory cells at the site of spinal cord injury, relieve cell apoptosis and lipid oxidation, improve vascular permeability of damaged tissues, and promote a recovery of neurological functions.^12^,^13^ The most unfavorable factors for recovery of spinal cord injury include lack of nerve growth factor (NGF), neuronal apoptosis, shedding of myelin sheath, and syringomyelia in an oxygen-deficient environment.^14^ Since spinal cord injury and post-injury recovery involve a variety of physio-pathological mechanisms, a targeted combination regime appears more beneficial in the treatment.

Both neurocyte and the central nervous system (CNS) are highly dependent on the presence of oxygen. It is especially true when tissue edema occurs after an injury. In such case, an effective improvement in oxygen supply is of high value for alleviating the severity of tissue damage and improving the prognosis. However, conventional oxygen inhalation cannot eliminate such pathological changes as anoxia and edema of spinal cord, thus it is of limited help in improving the prognosis.^15^,^16^ Hyperbaric oxygen therapy is highly valued in dealing with nerve injury.^17^ BDNF and BFGF in serum can reflect the severity of nerve damage. The higher they are, the more damaged the nerve is.^18^ On the other hand, a decrease in BDNF and BFGF indicates a relief in the injury and gradual recovery of nervous tissues. Our research findings suggest there are no significant differences in two cytokines: BDNF and BFGF between two groups before the treatment, but both cytokines are significantly reduced after the treatment. It means the injured spinal cord significantly recovers from the injury after treatment is applied, but the recovery is more evident in the experimental group than in the control group. The results indicate hyperbaric oxygen therapy plays an important role in the recovery from nerve injury and this has a positive meaning for the patients.

The diuretic mannitol can be used to reduce intracranial pressure, inhibit cell peroxidization and apoptosis, protect cellular tissues, and relieve secondary damage.^19^ As an important member of the inflammatory factor family, IL-6 represents the inflammatory response within human body. When its level declines, it means the inflammatory response gets reduced and improved. The present study confirms a significant decrease occurs in IL-6 level in experimental group when compared with the control group. There is evidence that mannitol can protect the nervous system very well. Similar effect has been witnessed in hyperbaric oxygen therapy. Thus, it can be speculated that those two therapies can exert synergistic effect when being combined.

Riluzole is a kind of benzothiazoles of neuroprotection effect. A large number of preclinical experiments have verified that riluzole can reduce the glutamate-mediated excitotoxicity and reduce cell apoptosis by blocking the voltage-gated Na + and Ca2 + pathways on the neuron membrane of presynaptic and postsynaptic neurons.^20^ Fehlings et al.^21^ has reported oral administration of 50mg Riluzole (bid, 14d) among the subjects in a perspective study. When compared with the control group, Riluzole group showed no sign of adverse effect or case of death. The movement score of 24 cervical cord-injured patients grew by 31.2 in average on 90 day after being admitted into the hospital, while the score growth in control group was 15.7 only (P=0.021). In present study, the experimental group had achieved significant progress in scores of both sensory and movement scores when compared with the control group (p<0.05). The outcome is in agreement with the previous report.

In general, the development and progression of thoracolumbar vertebral fracture-induced ASCI are affected by multiple factors, among which secondary injury plays a critical role in the prognosis of the disease. Therefore, apart from active operation for pressure reduction and fixation, application of pathophysiology-specific combined therapy can benefit the patients a lot. The regime of combining Riluzole with mannitol and hyperbaric oxygen therapy enjoys certain strengths when compared with conventional therapies due to its ability in significantly improve patients’ movement and sensory functions, alleviating inflammatory response of spinal cord, and facilitating a recovery from the injury.

## CONCLUSIONS

The patients receiving a combined therapy of Riluzole with mannitol and hyperbaric oxygen have achieved higher sensory and movement scores than those administered with conventional therapies alone. In the experimental group, levels of inflammatory factors in serum are significantly reduced, so are the cytokines that indicate neurocyte damage (BDNF and BFGF). It suggests combining Riluzole with mannitol and hyperbaric oxygen is able to inhibit inflammatory response of spinal cord and facilitate a recovery of the injured spinal cord. The combined therapy enjoys certain strengths over the conventional one, but it needs longer follow-up visit in order to further assess the long-term effect.

### Limitations of study

This study still has certain shortcomings, including insufficient number of cases and insufficient follow-up time. Therefore, larger sample size and longer follow-up visit are needed to verify the findings. As more novel reagents are developed and increasingly more microscopic exploration is made into the pathological and physiological courses of the disease, better therapies will be developed for sure.

### Authors’ Contributions:

**HL & JZT:** Designed this study and prepared this manuscript, and are responsible and accountable for the accuracy or integrity of the work;

**JC:** Collected and analyzed clinical data.

**JSF:** Significantly revised this manuscript.

## References

[1] Lee BB, Cripps RA, Fitzharris M, Wing PC (2014). The global map for traumatic spinal cord injury epidemiology:update 2011, global incidence rate. Spinal Cord.

[2] Mabray MC, Talbott JF, Whetstone WD, Dhall SS, Phillips DB, Pan JZ (2016). Multidimensional Analysis of Magnetic Resonance Imaging Predicts Early Impairment in Thoracic and Thoracolumbar Spinal Cord Injury. J Neurotrauma.

[3] Rogers WK, Todd M (2016). Acute spinal cord injury. Best Pract Res Clin Anaesthesiol.

[4] Kerwin AJ, Yorkgitis BK, Ebler DJ, Madbak FG, Hsu AT, Crandall ML (2018). Use of diaphragm pacing in the management of acute cervical spinal cord injury. J Trauma Acute Care Surg.

[5] Bydon M, Lin J, Macki M, Gokaslan ZL, Bydon A (2014). The current role of steroids in acute spinal cord injury. World Neurosurg.

[6] Burns SP, Tansey KE (2020). The Expedited International Standards for Neurological Classification of Spinal Cord Injury (E-ISNCSCI). Spinal Cord.

[7] Witiw CD, Fehlings MG (2015). Acute Spinal Cord Injury. J Spinal Disord Tech.

[8] Pili R, Gaviano L, Pili L, Petretto DR (2018). Ageing, Disability, and Spinal Cord Injury:Some Issues of Analysis. Curr Gerontol Geriatr Res.

[9] Eckert MJ, Martin MJ (2017). Trauma:Spinal Cord Injury. Surg Clin North Am.

[10] Mabray MC, Talbott JF, Whetstone WD, Dhall SS, Phillips DB, Pan JZ (2016). Multidimensional Analysis of Magnetic Resonance Imaging Predicts Early Impairment in Thoracic and Thoracolumbar Spinal Cord Injury. J Neurotrauma.

[11] Rouanet C, Reges D, Rocha E, Gagliardi V, Silva GS (2017). Traumatic spinal cord injury:current concepts and treatment update. Arq Neuropsiquiatr.

[12] Todd NV, Skinner D, Wilson-MacDonald J (2015). Secondary neurological deterioration in traumatic spinal injury:data from medicolegal cases [published correction appears in Bone Joint J 2015 97-B(12):1732]. Bone Joint J.

[13] Hachem LD, Ahuja CS, Fehlings MG (2017). Assessment and management of acute spinal cord injury:From point of injury to rehabilitation. J Spinal Cord Med.

[14] Tran AP, Silver J (2015). Neuroscience. Systemically treating spinal cord injury. Science.

[15] Rei M, Ayres-de-Campos D, Bernardes J (2016). Neurological damage arising from intrapartum hypoxia/acidosis. Best Pract Res Clin Obstet Gynaecol.

[16] Chen D, Huang X, Gan H, Du X, Lu S, Huang R (2017). Efficacy of alogliptin combined with motor imagery under hyperbaric oxygen in diabetic nephropathy with silent cerebral infarction. Biomed Rep.

[17] Baratz-Goldstein R, Toussia-Cohen S, Elpaz A, Rubovitch V, Pick CG (2017). Immediate and delayed hyperbaric oxygen therapy as a neuroprotective treatment for traumatic brain injury in mice. Mol Cell Neurosci.

[18] Robinson J, Lu P (2017). Optimization of trophic support for neural stem cell grafts in sites of spinal cord injury. Exp Neurol.

[19] Witherspoon B, Ashby NE (2017). The Use of Mannitol and Hypertonic Saline Therapies in Patients with Elevated Intracranial Pressure:A Review of the Evidence. Nurs Clin North Am.

[20] Martins BC, Torres BBJ, de Oliveira KM, Lavor MS, Osório CM, Fukushima FB (2018). Association of riluzole and dantrolene improves significant recovery after acute spinal cord injury in rats. Spine J.

[21] Fehlings MG, Nakashima H, Nagoshi N, Chow DS, Grossman RG, Kopjar B (2016). Rationale, design and critical end points for the Riluzole in Acute Spinal Cord Injury Study (RISCIS):a randomized, double-blinded, placebo-controlled parallel multi-center trial. Spinal Cord.

